# Melanogenesis Inhibitor(s) from *Phyla nodiflora* Extract

**DOI:** 10.1155/2012/867494

**Published:** 2012-11-12

**Authors:** Feng-Lin Yen, Moo-Chin Wang, Chan-Jung Liang, Horng-Huey Ko, Chiang-Wen Lee

**Affiliations:** ^1^Department of Fragrance and Cosmetic Science, College of Pharmacy, Kaohsiung Medical University, Kaohsiung 80708, Taiwan; ^2^Department of Anatomy and Cell Biology, College of Medicine, National Taiwan University, Taipei 10063, Taiwan; ^3^Department of Nursing, Division of Basic Medical Sciences, Chronic Diseases and Health Promotion Research Center, Chang Gung Institute of Technology, Chia-Yi 61363, Taiwan

## Abstract

Overexpression of tyrosinase can cause excessive production of melanin and lead to hyperpigmentation disorders, including melasma and freckles. Recently, agents obtained from plants are being used as alternative medicines to downregulate tyrosinase synthesis and decrease melanin production. *Phyla nodiflora* Greene (Verbenaceae) is used as a folk medicine in Taiwanese for treating and preventing inflammatory diseases such as hepatitis and dermatitis. However, the antimelanogenesis activity and molecular biological mechanism underlying the activity of the methanolic extract of *P. nodiflora* (PNM) have not been investigated to date. Our results showed that PNM treatment was not cytotoxic and significantly reduced the cellular melanin content and tyrosinase activity in a dose-dependent manner (*P* < 0.05). Further, PNM exhibited a significant antimelanogenesis effect (*P* < 0.05) by reducing the levels of phospho-cAMP response element-binding protein and microphthalmia-associated transcription factor (MITF), inhibiting the synthesis of tyrosinase, tyrosinase-related protein-1 (TRP-1), and TRP-2, and decreasing the cellular melanin content. Moreover, PNM significantly activated the phosphorylation of mitogen-activated protein kinases, including phospho-extracellular signal-regulated kinase, c-Jun N-terminal kinase, and phospho-p38, and inhibited the synthesis of MITF, thus decreasing melanogenesis. These properties suggest that PNM could be used as a clinical and cosmetic skin-whitening agent to cure and/or prevent hyperpigmentation.

## 1. Introduction

Melanogenesis is a well-known mechanism for preventing skin damage caused by ultraviolet (UV) radiation [1]. However, overproduction of melanin may be caused by overexposure to UV rays, inflammation, and many skin injuries and may result in many disorders of hyperpigmentation such as melasma and freckles [2, 3]. Hyperpigmentation is commonly observed in patients with skin types IV and V, especially in Asian and Indian population. Hyperpigmentation not only causes aesthetic problems, such as skin discoloration, but also has a significant impact on the psychological status of an individual, for example, this condition may decrease social functioning, reduce productivity at work or school, and lower the self-esteem of patients [4–6].

Tyrosinase-related proteins (TRPs) such as tyrosinase (TYR), TRP-1 (5,6-dihydroxyindole-2-carboxylic acid oxidase), and TRP-2 (dopachrome tautomerase) are rate-limiting enzyme in the process of melanogenesis. TYR hydroxylates tyrosine to dihydroxyphenylalanine (DOPA) and oxidizes DOPA to the corresponding dopaquinone. In turn, TRP-2 catalyzes the conversion of dopachrome to 5,6-dihydroxyindole-2-carboxylic acid (DHICA) and TRP-1 oxidizes the DHICA to indole-5,6-quinone carboxylic acid and subsequently produces melanin [7]. Hence, overactivity of TRPs can cause abnormal accumulation of melanin pigments and lead to hyperpigmentation disorders such as melasma and lentigo senilis [8]. Moreover, microphthalmia-associated transcription factor (MITF), a basic helix-loop-helix leucine zipper transcription factor involved in the development of melanocytes, is a major regulator of the synthesis of TRPs [9, 10]. Previous studies have shown that cyclic adenosine monophosphate response element-binding protein (CREB) can bind to the cAMP response element motif of the MITF promoter and increase the level of MITF protein. Therefore, phosphorylation of CREB can regulate the expression of tyrosinase and induce melanogenesis through MITF transcription [11, 12]. In addition, the phosphorylation of mitogen-activated protein kinases (MAPKs) such as extracellular signal-regulated kinase (ERK), c-Jun N-terminal kinase (JNK), and p38 and effectively modulates the transcription of MITF, thereby leading to antimelanogenesis [13–15].

*Phyla nodiflora* (L) Greene (Verbenaceae), a Taiwanese folk medicine, has been widely used as an herbal drink, and a nourishing agent and an immunomodulator and anti-inflammatory agent to prevent many diseases [16]. *P*. *nodiflora* and *Lippia nodiflora* are synonyms, and *P*. *nodiflora* possesses many pharmacological effects such as antiseptic, antitussive, antipyretic, and anti-inflammatory [17–19]. Abbasi et al. have also mentioned the ethnopharmacological application of *P*. *nodiflora* for skin diseases and in folk cosmetics, such as pimples, carbuncle, and skin burns [20]. The phytochemical ingredients of *P*. *nodiflora*, including flavonoids (hispidulin, eupafolin, Nodifloretin-A) [21, 22], flavone glycosides (lippiflorin A and B) [23], alkaloids, essential oils (methyl salicylate, eugenol), resin (*α*-copaene, *β*-bisabolene) [24], quinol (halleridone and hallerone) [25], and steroidal (4′,5′-dimethoxybenzoloxystigmasterol, *γ*-sitosterol) [26, 27], have been previously identified. These phytochemicals are suggested to be responsible for the pharmacological effects of *P*. *nodiflora*. In addition, the ethanol extract of *P*. *nodiflora* exerts antiurolithiatic activity by reducing the supersaturation of urine with calcium oxalate and has diuretic properties and antioxidant potential [28]. Balamurugan et al. have demonstrated that *γ*-sitosterol isolated from the methanol extract of* P*. *nodiflora* had antidiabetic activity to prevent the streptozotocin induced diabetic [27]. Ahmed et al. have also manifested that the methanol extract of *P*. *nodiflora* had anti-inflammatory and antinociceptive effects [29]. These reports indicated that the active phytochemicals are easily extracted with organic solvents such as methanol and ethanol. However, the antimelanogenesis activity and molecular biological mechanism of *P*. *nodiflora* has not been investigated to date. The present study was therefore used methanol to extract the *P*. *nodiflora* for evaluating its antimelanogenesis activity.

This study aimed to determine the effect on melanin production and biological mechanisms underlying antimelanogenesis of methanol extract of *P*. *nodiflora* (PNM) in B16F10 cells. We determined cell viability, cellular melanin content, and tyrosinase activity for estimating the decrease in melanin production. In addition, western blotting was performed to determine the expression of tyrosinase regulator (p-CREB and MITF); TRPs (TYR, TRP-1, and TRP-2); and agents responsible for MITF degradation (p-ERK, p-JNK, and p-p38) for elucidating the biological mechanism of antimelanogenesis.

## 2. Materials and Methods

### 2.1. Chemicals and Reagents

B16F10 melanoma and 3T3 (mouse embryonic fibroblast) cells were purchased from Bioresource Collection and Research Center (BCRC, Hsinchu, Taiwan). Dimethyl sulfoxide (DMSO), 3-(4,5-dimethylthiazol-2-yl)-2,5-diphenyltetrazolium bromide (MTT), and L-DOPA were purchased from Sigma-Aldrich Chemicals Co. (St. Louis, MO, USA). The antibodies for phospho-ERK (p-ERK) (Thr202/Tyr204), p-p38 (Thr180/Tyr182), p-JNK (Thr183/Tyr185), and p-CREB (Ser 133) were purchased from Cell Signaling Technology (USA). MITF, TYR, TRP1, TRP-2, GAPDH, anti-mouse, anti-goat, and anti-rabbit horseradish peroxidase-conjugated immunoglobulin G (IgG) antibodies were purchased from Santa Cruz Biotechnology (USA).

### 2.2. Preparation of PNM

We collected *P*. *nodiflora* from a local farm (Tainan, Taiwan) in April 2007. The authenticity of the plant species was identified by a pharmacognosist, Professor Chen, and stored as a voucher specimen (2007-02-PNM) in the Herbarium of the Department of Fragrance and Cosmetic Science, Kaohsiung Medical University, Kaohsiung, Taiwan. We powdered 200 g of the dried aerial part of *P*. *nodiflora* and immersed them in a flask with 1 L of methanol and then decocted (boiled under reflux) this mixture for 2 h; this extraction procedure was repeated 3 times. The entire methanolic extract of *P*. *nodiflora* was blended and filtered using filter paper. Then, the filtrate was concentrated by rotary vacuum evaporation and then lyophilized in a freeze dryer and calculated. The method can obtain 28.2 grams of freeze-dried methanol extract and the yield calculated for crude methanol extract was 14.1% with respect to the initial dry material. The dry powders of PNM were placed at −20°C until use.

### 2.3. Cell Viability after Treatment with PNM

The viability of cells treated with PNM was determined according to the method of Ye et al. [30]. B16F10 and 3T3 (mouse embryonic fibroblast) cells were, respectively, cultured in Dulbecco's modified Eagle's medium (DMEM; Gibco Life Technologies, Carlsbad, CA, USA) supplemented with 10% fetal bovine serum, 100 units/mL penicillin G, 100 *μ*g/mL streptomycin, and 0.25 *μ*g/mL amphotericin and then were incubated at 37°C with 5% CO_2_. The viability of cells treated with PNM was determined by MTT assay. Briefly, 1 × 10^4^ B16F10 or 3T3 cells were seeded and adhered in 96-well plates. After 24 h, DMEM was removed and 90 *μ*L of fresh DMEM and 10 *μ*L of different concentrations of PNM were added, and the cells were incubated for 48 h. We used 1% DMSO as a control for comparing the viability of cells treated with PNM. After 48 h incubation, the medium was removed, and the cells were washed twice with phosphate-buffered saline; further, 150 *μ*L of MTT in DMEM solution (0.5 mg/mL) was added to each well, and the cells were incubated for 4 h at 37°C. Subsequently, the MTT solution was removed and 100 *μ*L of DMSO was added into each well, and the plate was gently shaken for dissolving the formazan crystals. The absorbance of each well was measured at 550 nm using a microplate spectrophotometer (BIOTEK, *μ*Quant). The absorbance of 1% DMSO was used as a control to compare the absorbance of cells treated with different concentrations of PNM. All determinations were performed in triplicate.

### 2.4. Determination of Cellular Melanin Content

Cellular melanin content was determined as described previously [30]. Briefly, 1 × 10^5^ B16F10 cells were seeded in 6-well plates and cultured at 37°C for 24 h. Subsequently, the cells were treated with 1% DMSO and different concentrations of PNM for 48 h. We used 1% DMSO as control. Then, the cells were washed with PBS and lysed in 150 *μ*L of 1 M NaO at 95°C. We added 100 *μ*L of the lysate in a well of a 96-well microplate and quickly measured the absorbance at 490 nm using a microplate spectrophotometer (BIOTEK, *μ*Quant). All determinations were performed in triplicate.

### 2.5. Determination of Cellular Tyrosinase Activity

Cellular tyrosinase activity was measured by the method of Tsang et al. with some modification [31]. Briefly, the culture method for determining cellular tyrosinase assay was similar to that for determining melanin content. After treatment with different concentration of PNM 48 h, the cells were collected after treatment with trypsin-EDTA and centrifuged at 12,000 rpm for 10 min to obtain cell pellets. The pellets were lysed with 100 *μ*L 1% Triton X-100 and 100 *μ*L of 0.1 mM PBS (pH 6.8) containing phenylmethylsulfonyl fluoride. The pellet solutions were frozen and thawed twice and then centrifuged at 12,000 rpm for 10 min. We added 80 *μ*L of the supernatant in a 96-well plate and mixed with 20 *μ*L of 0.2% L-DOPA. After incubation for 1 h, the optical densities were measured at 475 nm using a microplate spectrophotometer (BIOTEK, *μ*Quant). The inhibitory activity of the PNM-treated cells was presented as percentage against that of the untreated cells.

### 2.6. Analysis of the Expression of Proteins Regulating Melanogenesis by Western Blotting

B16F10 cells were treated with different concentrations of PNM. Cells were collected and lysed in a sample buffer containing 4% sodium dodecyl sulfate (SDS), 20% glycerol, 10% 2-mercaptoethanol, 0.004% bromphenol blue, and 0.125 M Tris HCl. The lysates as protein samples were denatured at 95°C for western blot assay. Proteins were separated using 12% SDS-polyacrylamide gel electrophoresis (SDS-PAGE) running gel. Subsequently, the resolved proteins were transferred to nitrocellulose membranes and then were blocked using 5% dried milk in Tris HCl buffer. Membranes were incubated with different primary antibodies for 24 h, including MITF, p-CREB, TYR, TRP1, TRP-2, p-ERK, p-p38, p-JNK, and GAPDH, and were further incubated with anti-mouse or anti-rabbit horseradish peroxidase antibody for 1 h. The bands of bound antibodies were detected by enhanced chemiluminescence reagents, and the images of protein expression were obtained using an AlphaImager HP High Resolution Imaging System. All determinations were performed in triplicate.

### 2.7. Statistical Analysis

All data are expressed as means ± standard deviations of the indicated number of experiments. Data were analyzed by one-way ANOVA followed with Tukey's post-hoc test to calculate statistical significance using the SPSS software (Version 19). *P* < 0.05 was considered to indicate a significant difference.

## 3. Results

### 3.1. Viability of Cells after Treatment with PNM

**Figure 1:**
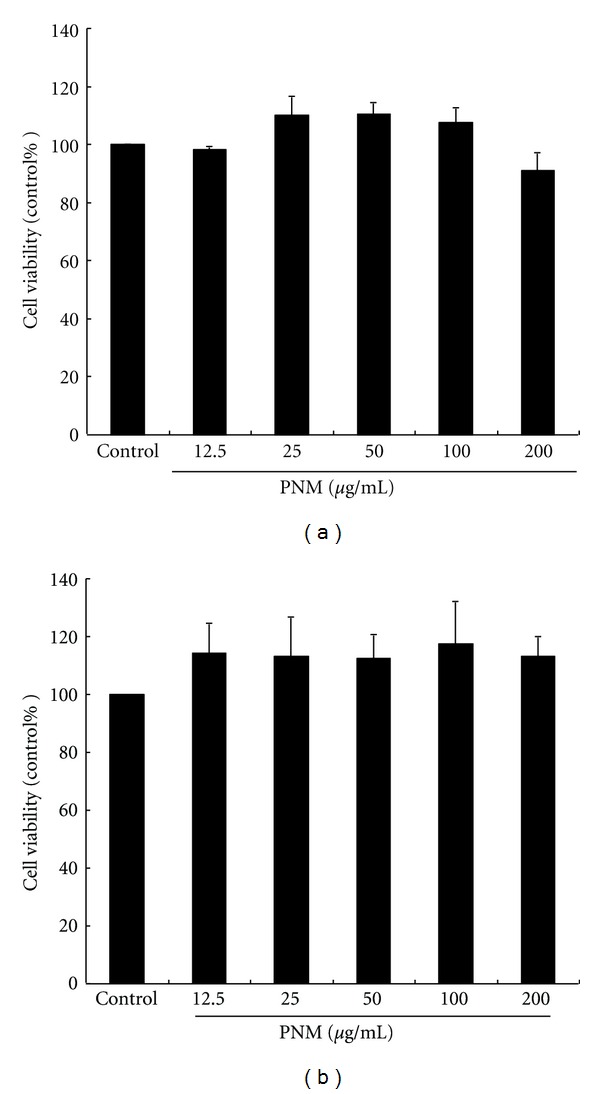
The viability of B16F10 (a) and 3T3 (b) cells treated with a methanolic extract of *Phyla nodiflora*.

In vitro safety of the extract or pure compound is the first consideration in formulating an agent as a health food and/or cosmetic. We determined the cytotoxicity of PNM in B16F10 cells by an MTT assay. The viability of B16F10 cells treated with different concentrations of PNM is shown in Figure 1(a). B16F10 cells were treated with a serial dose of PNM (12.5 to 200 *μ*g/mL), and their viability was more than 90%. In addition, the viability of 3T3 cells treated with the same concentration of PNM was not less than 90% (Figure 1(b)). These results indicated that PNM is a safe ingredient for determining the antimelanogenesis effect of PNM. Therefore, we used PNM at doses of 12.5–100 *μ*g/mL to determine the cellular melanin synthesis and tyrosinase activity in B16F10 cells.

### 3.2. Cellular Melanin Synthesis and Inhibition of Tyrosinase Activity by PNM

**Figure 2:**
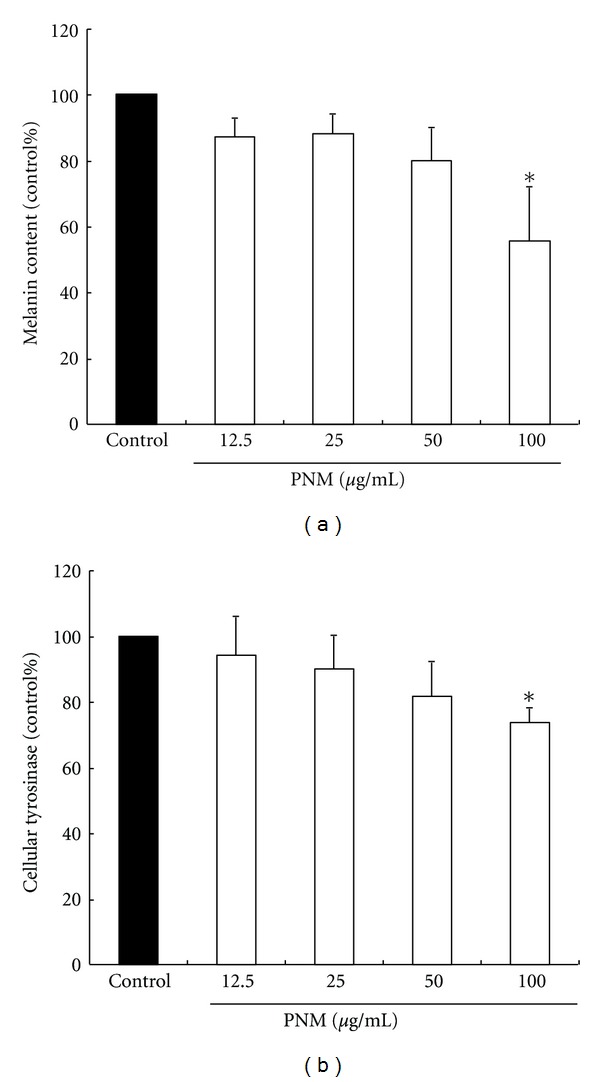
The cellular melanin content (a) and tyrosinase activity (b) in B16F10 cells treated with a methanolic extract of *Phyla nodiflora*. The different superscript letters indicate significant difference at *P* < 0.05 using ANOVA followed with Tukey's post-hoc test. *Significantly different from control.

**Figure 3:**
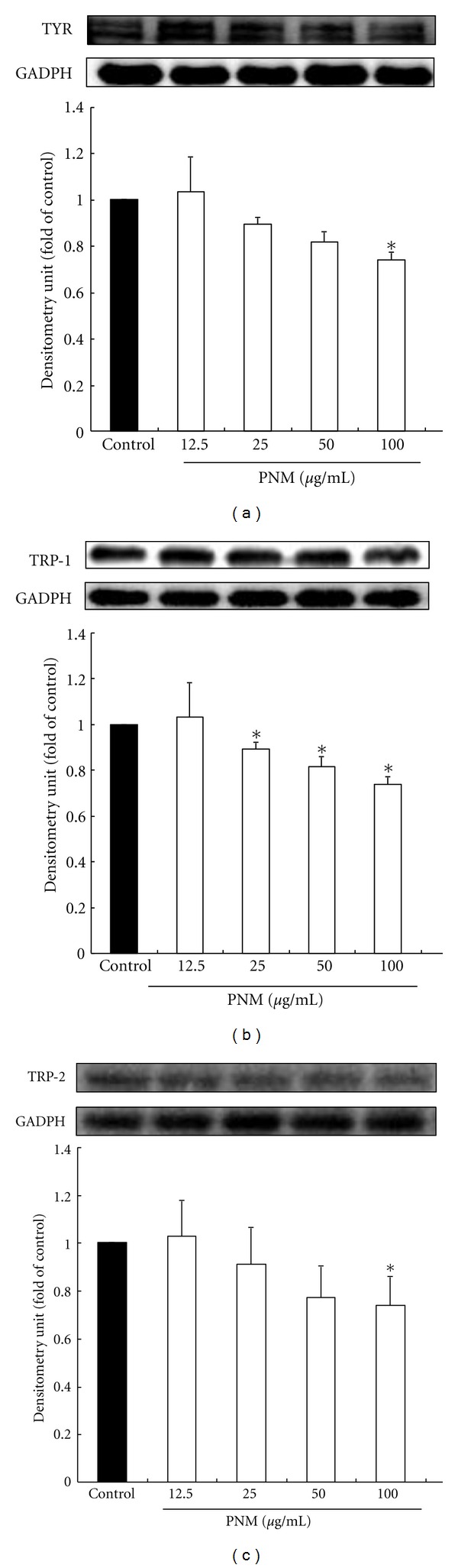
Methanolic extract of *Phlya nodiflora* decreases the melanin production by inhibiting the levels of tyrosinase-related proteins (TRPs) (a) tyrosinase (TYR), (b) TRP-1, and (c) TRP-2. The different superscript letters indicate significant difference at *P* < 0.05 using ANOVA followed with Tukey's post-hoc test. *Significantly different from control.

Cellular tyrosinase activity is the major factor that stimulates melanin synthesis and ultimately induces melanogenesis [7]. We determined the cellular tyrosinase activity and melanin content for investigating the antimelanogenesis activity of PNM on B16F10 cells. B16F10 cells were pretreated with PNM at dose of 12.5–100 *μ*g/mL (Figure 2(a)). PNM treatment significantly decreased the cellular melanin content in a dose-dependent manner compared to that in the control group (*P* < 0.05). In addition, PNM treatment significantly reduced the cellular tyrosinase activity in a dose-dependent manner compared to the control (*P* < 0.05) (Figure 2(b)). Further, we determined the protein level of TYR, TRP-1, and TRP-2 by western blotting. PNM treatments effectively reduced the synthesis of TYR (Figure 3(a)), TRP-1 (Figure 3(b)), and TRP-2 (Figure 3(c)), thereby inhibiting melanogenesis in a dose-dependent manner. These results indicate that PNM treatment reduced the melanin content and inhibited cellular tyrosinase activity in B16F10 cells.

### 3.3. PNM Reduces the Synthesis of Tyrosinase by Inhibiting MITF and p-CREB Proteins

**Figure 4:**
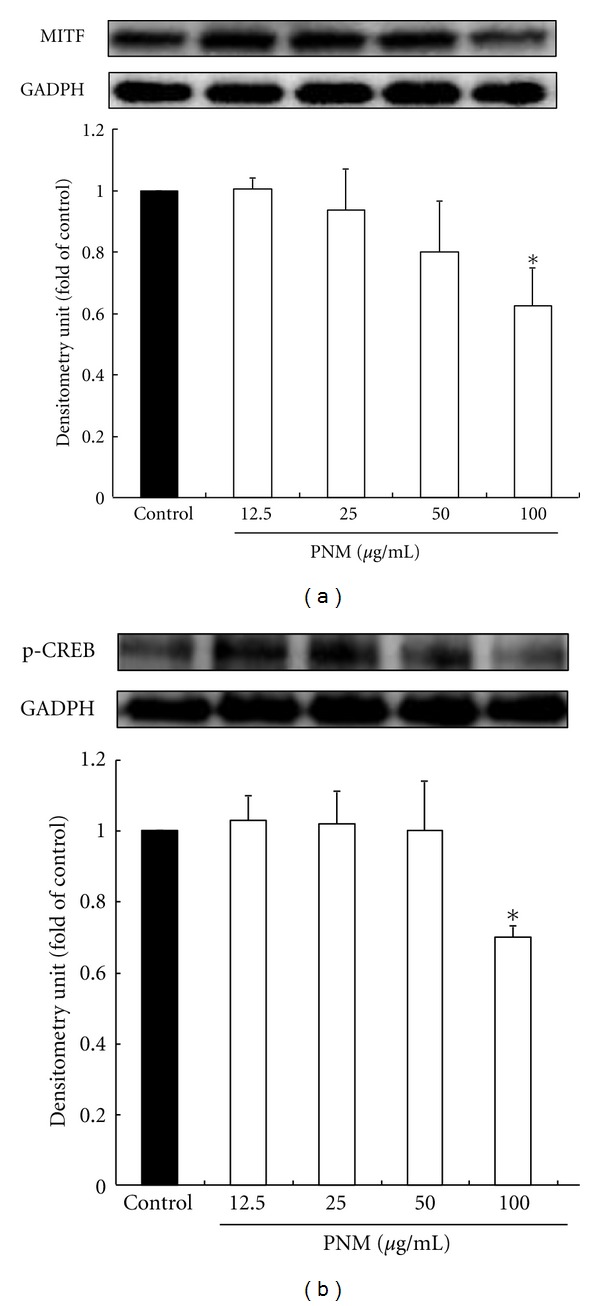
Methanolic extract of *Phyla nodiflora* downregulates the expression of (a) microphthalmia-associated transcription factor (MITF) and phospho-cyclic adenosine monophosphate response element-binding protein (p-CREB) (b). The different superscript letters indicate significant difference at *P* < 0.05 using ANOVA followed with Tukey's post-hoc test. *Significantly different from control.

Synthesis of the protein tyrosinase is closely regulated by MITF and p-CREB proteins, including TYR, TRP-1, and TRP-2, which leads to melanogenesis [12–15]. Therefore, we performed western blotting to determine the expression levels of MITF and p-CREB in B16F10 cells treated with serial doses of PNM (12.5–100 *μ*g/mL). PNM treatments significantly reduced the expression levels of MITF (Figure 4(a)) and p-CREB (Figure 4(b)) in B16F10 cells in a dose-dependent manner. These findings clearly indicated that the antimelanogenesis effect of PNM is directly related to reduced synthesis of tyrosinase by downregulation of the expression of MITF and p-CREB.

### 3.4. PNM Inhibits Melanogenesis by Degradation of MITF via MAPK Signaling Pathway

**Figure 5:**
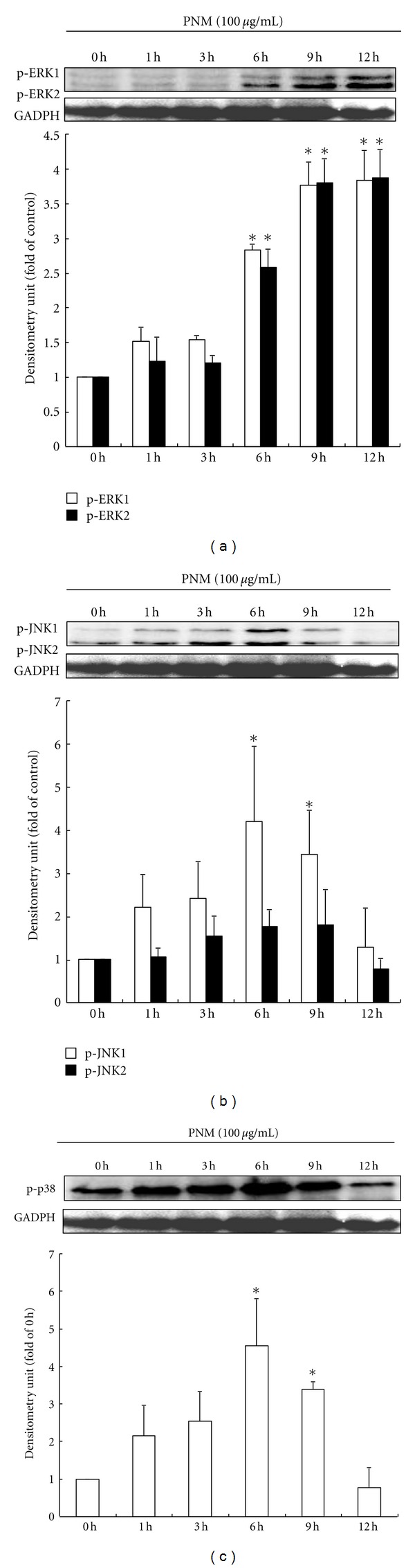
Methanolic extract of *Phyla nodiflora* degrades the MITF protein by activating phosphorylation of mitogen-activated protein kinases (MAPKs), (a) phospho-extracellular signal-regulated kinase (ERK), (b) phosphor-c-Jun N-terminal kinase (p-JNK), and (c) p-p38. The different superscript letters indicate significant difference at *P* < 0.05 using ANOVA followed with Tukey's post-hoc test. *Significantly different from 0 h.

**Figure 6:**
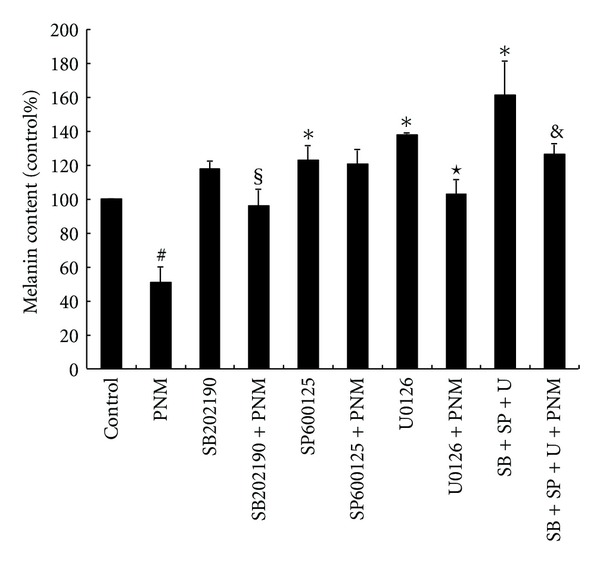
Methanolic extract of *P*. *nodiflora* decreased the melanin production induced by mitogen-activated protein kinase (MAPK)-specific inhibitors. The different superscript letters indicate significant difference at *P* < 0.05 using ANOVA followed with Tukey's post-hoc test. ^#^Significant different from control; *U0126, SB202190, SP600125, and treatment with a combination of all 3 inhibitors compared to control; ^§^PNM treated with SB202190 inhibitor compared to SB202190 only. ^*⋆*^PNM treated with U0126 inhibitor compared to U0126 only. ^&^PNM treated with a combination of all 3 inhibitors compared to treatment with all 3 inhibitors.

Previous studies have shown that phosphorylation of MAPKs such as ERK, JNK, and p38 mainly inhibit the synthesis of MITF and thus reduce the levels of tyrosinase, thereby inhibiting melanogenesis [13–15]. Therefore, western blotting was performed to determine the effect of 100 *μ*g/mL of PNM on the expression level of p-ERK, p-JNK, and p-p38 in a time-course experiment. Our results showed a marked increase in the expression of p-ERK (Figure 5(a)), p-JNK (Figure 5(b)), and p-p38 (Figure 5(c)) after treatment with 100 *μ*g/mL of PNM at 12, 6, and 6 h, respectively, (*P* < 0.05). These data indicate that PNM may induce the phosphorylation of 3 MAPKs and subsequently change the degradation of levels of MITF protein. In addition, we further investigated whether the effect of PNM treatment on melanin synthesis is prevented by the addition of 10 *μ*M of U0126 (a selective inhibitor of MAPK/ERK), SB202190 (a selective inhibitor of p38), SP600125 (a selective inhibitor of JNK), and a combination of all inhibitors. Addition of each inhibitor increased the melanin production and showed a significant difference (*P* < 0.05) compared to that in the control group (Figure 6). The treatment with a combination of all inhibitors significantly increased the melanin production compared to that in the control group and in the group treated with a single inhibitor (*P* < 0.05). Our results showed that the melanin production in cells treated with U0126, SB202190, and a combination of all inhibitors in the presence of 100 *μ*g/mL of PNM was significantly lower (*P* < 0.05) than that in cells treated only with the inhibitor; however, this effect is not significant in the case of cells treated with SP600125. Thus, these results suggest that PNM exerts antimelanogenesis effects through phosphorylation of both ERK and p38 but not of JNK.

## 4. Discussion

Safety is the first and foremost consideration while developing therapeutic or cosmetic agents using active ingredients obtained from plants. Hydroquinone is a commonly used skin-whitening agent for treating and preventing hyperpigmentation disorders such as melasma and freckles. However, previous studies have shown that hydroquinone itself can cause a depigmentation of the skin, for example, vitiligo, as a side effect because of melanocyte cytotoxicity. Therefore, use of hydroquinone as an additive in a cosmetic or skin care product is prohibited [32, 33]. According to that, we performed the cell viability of PNM for estimating the in vitro safety, including B16F10 and 3T3. Our data showed that PNM at a dose of 12.5–200 *μ*g/mL did not have any significant cytotoxicity in mouse embryonic fibroblast cells. These results indicated that PNM is a safe component and therefore the doses of PNM in the above range are safe for determining cellular melanin content, tyrosinase activity, and antimelanogenesis effect.

TRPs are rate-limiting enzymes in the melanogenesis process that increase the conversion of tyrosine to dopaquinone, the rearrangement of dopachrome to DHICA and thus cause overproduction and accumulation of melanin pigments in the skin. In our study, PNM treatment inhibited the cellular tyrosinase activity in a dose-dependent manner and thus reduced the melanin content in B16F10 cells, especially at a dose of 100 *μ*g/mL of PNM. In addition, 200 *μ*M of kojic acid did not decrease the melanin content in B16F10 cells (data not shown). Hyperpigmentation is by overactivity of TRPs, but not all skin-whitening agents can simultaneously inhibit TYR, TRP-1, and TRP-2, such as *Viola mandshurica* [34] and nicotinic acid hydroxamate [35]. However, our western blotting assay showed that PNM treatment reduced the expression of all rate-limiting enzymes, including TYR, TRP-1, and TRP-2 protein, and prevented abnormal accumulation of melanin in the process of melanogenesis. These data suggested that the decrease in melanin content by PNM treatment was because of inhibition of TRPs; therefore, PNM could be used as a skin-whitening agent against hyperpigmentation.

MITF is the major regulator of the synthesis of TRPs such as TYR, TRP-1, and TRP-2 during the process of melanogenesis in mammalian cells [16–18]. Our result showed that cells treated with PNM showed degradation of the MITF protein in a dose-dependent manner compared to the cells in the control group (*P* < 0.05); therefore, PNM treatment can inhibit the synthesis of the proteins TYR, TRP-1, and TRP-2 through upregulation of MITF degradation. Moreover, phosphorylation of CREB is a prime factor that interacts with the cAMP response element motif of the MITF in the cAMP pathway, which stimulates tyrosinase synthesis and in turn the synthesis of melanin [10, 11]. Our results indicated that PNM treatment showed a dose-dependent reduction in the expression level of p-CREB level to inhibit the tyrosinase synthesis and melanin production. A recent study showed that a traditional Chinese medicine, Qian-wang-hong-bai-san, downregulated the expression level of p-CREB and MITF to inhibit tyrosinase synthesis and melanin production in B16 cells [31]. These results indicate that PNM is a good skin-whitening agent, and it inhibits tyrosinase synthesis and decreases the production of melanin by inhibiting phosphorylation of CREB and degradation of MITF.

On the other hand, activation of MAPK signaling pathway plays a role in the phosphorylation of MITF at serine-73 and subsequently leads to ubiquitination of MITF followed by proteasome-mediated degradation and thus inhibits tyrosinase synthesis and melanin production [12–15]. Previous studies have shown that skin-whitening agents such as *Cuscuta japonica* [36] and quercetin [37] activate the phosphorylation of MAPKs and downregulate the expression of MITF and subsequently inhibit the synthesis of TRPs and melanin production. Our study indicated that PNM treatment significantly phosphorylated the MAPK proteins, including ERK, JNK, and p38, to degrade the MITF protein and further diminished tyrosinase, TRP-1, and TRP-2 synthesis for decreasing melanin production. Furthermore, the inhibitors of ERK, JNK, and p38 were added in B16F10 cells treated with PNM to confirm the intracellular signaling pathway regulating melanin production. Our results indicated that PNM could reduce the overproduction of melanin after addition of specific inhibitors of ERK and p38; therefore, PNM-mediated decrease in melanin production was thought to occur via activation of ERK and p38 pathways and subsequent degradation of MITF protein to inhibit tyrosinase synthesis in B16F10 cells.

In conclusion, the present study is firstly demonstrated that PNM treatment inhibits melanogenesis by activating ERK and p38 signaling pathways, which lead to a downregulation of the MITF protein and finally reduces the synthesis of tyrosinase and production of melanin. Thus, on the basis of the molecular biological mechanism of PNM, we suggest that PNM can be safely used as be a skin-whitening agent, and we will perform clinical trials in the future to establish this treatment as evidence-based medicine and/or cosmetic.
